# Cell Nanomechanics Based on Dielectric Elastomer Actuator Device

**DOI:** 10.1007/s40820-019-0331-8

**Published:** 2019-11-11

**Authors:** Zhichao Li, Chao Gao, Sisi Fan, Jiang Zou, Guoying Gu, Mingdong Dong, Jie Song

**Affiliations:** 1 Institute of Nano Biomedicine and Engineering, Department of Instrument Science and Engineering, School of Electronic Information and Electrical Engineering, Shanghai Jiao Tong University, Shanghai, 200240 People’s Republic of China; 2 Shanghai Engineering Research Center of Advanced Dental Technology and Materials, Shanghai Ninth People’s Hospital, Shanghai Jiao Tong University, Shanghai, 200240 People’s Republic of China; 3 Robotics Institute, School of Mechanical Engineering, Shanghai Jiao Tong University, Shanghai, 200240 People’s Republic of China; 4 State Key Laboratory of Mechanical System and Vibration, Shanghai Jiao Tong University, Shanghai, 200240 People’s Republic of China; 5 Interdisciplinary Nanoscience Center (iNANO), Aarhus University, Aarhus, 8000 Denmark

**Keywords:** Dielectric elastomer actuator, Mechanical stimulus, Bioreactor, Mechanobiology

## Abstract

As a frontier of biology, mechanobiology plays an important role in tissue and biomedical engineering. It is a common sense that mechanical cues under extracellular microenvironment affect a lot in regulating the behaviors of cells such as proliferation and gene expression, etc. In such an interdisciplinary field, engineering methods like the pneumatic and motor-driven devices have been employed for years. Nevertheless, such techniques usually rely on complex structures, which cost much but not so easy to control. Dielectric elastomer actuators (DEAs) are well known as a kind of soft actuation technology, and their research prospect in biomechanical field is gradually concerned due to their properties just like large deformation (> 100%) and fast response (< 1 ms). In addition, DEAs are usually optically transparent and can be fabricated into small volume, which make them easy to cooperate with regular microscope to realize real-time dynamic imaging of cells. This paper first reviews the basic components, principle, and evaluation of DEAs and then overview some corresponding applications of DEAs for cellular mechanobiology research. We also provide a comparison between DEA-based bioreactors and current custom-built devices and share some opinions about their potential applications in the future according to widely reported results via other methods.

## Highlights

- The main components, principle, and technology of dielectric elastomer actuator (DEA) were reviewed to illustrate that DEA can be an effective carrier for mechanobiology research.
- Comparison between DEA-based bioreactors and current commercial devices is provided, as well as the outlook of the DEA bio-applications in the future.

## Graphical abstract

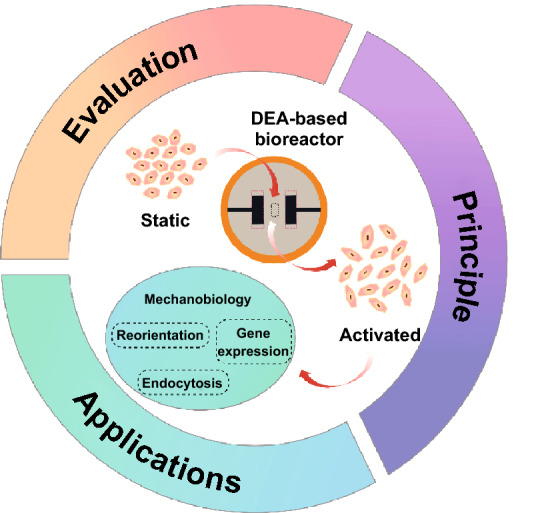

## Introduction

Mechanobiology is an emerging science concern about the effects of mechanical loadings and physical forces on cell behaviors and diseases [1]. Biological cells and tissues living in vivo environment are exposed to several mechanical stimulations such as stretching and contracting. As reported so far, mechanical loadings can be sensed by cells and then influence cellular behaviors like migration, proliferation, orientation, and gene expression [2–9]. Additionally, some diseases such as atherosclerosis [10] and cancers [11] are proved to have similar relation with mechanical cues as well. For example, Kim et al. [12] found that mechanical effects can affect cellular remodeling and regeneration of tissue, which means the possibility of developing cell-based therapies. Besides, Park et al. [13] confirmed that equiaxial and uniaxial strains have different induction effects on the differentiation of mesenchymal stem cells. In a word, people are now trying to achieve better understand of cellular mechanism for the purpose of developing more effective and advanced biomedical technology.

However, studying mechanical stimulus in vitro directly remains difficult because the traditional cell culture technology cannot provide such mechanics, so the first problem need to solve is to apply mechanical stimulations to cells while cultured in vitro to mimic the true environment in vivo. As reported so far, some methods have been taken to apply mechanical loadings on cells, including hydrostatic pressure and fluid shear [14–16], other interesting devices such as biochip [17], wrinkled skin-on-a-chip [18], pneumatic stretching system [19], motor-driven system [20], piezoelectric [21], and optical actuation methods [22, 23]. More recently, a custom-built open-source stretch system assembled from laser-cut acrylic plates emerged [24]. In general, such a gap between engineering and biology is attractive and even profitable; some companies have entered this market and are selling their products, which include Flexcell system [25, 26] from Bio-Equip, STB-1400 [27] from Strex, etc. Generally, these means require complex designs and result in complicated system structures and besides high costs. In contrast, dielectric elastomer actuators (DEAs) are simpler, have advantages of highly controllable deformation, sub-millisecond response time, and optically transparent, and can be integrated with cell culture environment conveniently. Since Pelrine et al. [28] presented their landmark discovery about electrostatically activated elastomeric actuators in 2000, this novel technology has been used in many fields, such as energy harvesters [29], tactile displays [30], soft robotics [31, 32] and what to be emphasized here, mechanically biological cells stimulus, and the potential application as the biosensor to measure the cellular contraction force.

In this review, firstly we introduce the basic dielectric elastomer actuator technology simply, including the components and actuation mechanism of the DEA devices, and the characterization methods. Secondly, we overview the applications of DEA-based devices in the field of cellular mechanical loading, which can be divided into bio-stretching device and biosensor. Thirdly, comparisons of popular commercial methods and DEA-based devices are made. Lastly, we further provide our prospect on DEAs’ applications in the future mechanobiology research.

## Components of the DEA Devices

DEAs are typically simple in structure and require very different materials with traditional actuators like electric motors. Briefly, the dielectric elastomer membrane (DEM) and the compliant electrodes are what demanded, the pre-stretched DEM sandwiched by electrodes [32, 33] and then fixed by rigid frames. These two main components are the key to determine the performance of DEA-based devices and combine with various pre-stretch sets to make DEA forms diversify.

### Dielectric Elastomer Membrane

The DEM belongs to one subcategory of electroactive polymers (EAPs), which can respond to electrical stimulation with significant size or shape change, and has already emerged as a new actuation material [34]. As one of the most important components of DEAs, the material properties of DEM directly determine the actuation performance of DEAs. Since the 1990s, researchers have conducted massive experiments to find proper DEM materials, such as silicones, polyurethanes, acrylics, and nitrile rubbers. Among these, silicones and acrylics are the two most commonly used materials. The most widely used acrylic DEM is 3 M VHB 4910 and 3 M VHB 4905 [35]. Both of them are made of a mixture of aliphatic acrylate, which shows a property of high viscosity, flexibility, and tensile resistance. However, the VHB-based DEAs show serious viscoelastic nonlinearity that makes the precision tracking control challenge [36–41].

Silicone rubber, which has good elastic properties, has fast strain response speed, and can maintain constant modulus at higher temperature, is one of the commonly used matrices for the preparation of DEM materials although the deformation degree of silicone membrane is low. Because of its weak viscoelastic characteristics, the response speed of silicone film is faster and shows higher efficiency. Besides, Akbari et al. [42] have presented theoretical guidelines for improving the deformation actuation of silicon-based DEM by changing the pre-stretch ratios.

Actually, DEA devices used for biomedical and bioinspired systems have already been reported [43], such as refreshable braille displays for the blinds and bioinspired tunable lenses for the visually impaired. However, as reported by Herbert Shea and colleagues, for cell- and tissue-related applications, the DEM materials to be chosen should satisfy some special requirements [44]: Firstly, they should be non-cytotoxic and compatible with standard cell culture protocols like sterilization and incubation; secondly, they need to be optically transparent for the convenience of integrating with the optical microscopes. After that, the selection of DEMs can be flexible since various designs and fabrications may be chosen. For example, some works used Sylgard 186 (Dow Corning) as the DEM and covered it with Silbione LSR 4305 (BlueStar Silicones) as the biocompatible membrane, which contacts the biological samples directly [44, 45]. Besides, as the alternative, other PDMS has been used as well [46–48]. In our group, ELASTOSIL Film 2030 250/100 from WACKER was used to meet the principles above.

### Materials and Techniques for Electrodes

Another indispensable element for DEAs is the compliant electrode; well-designed electrodes patterning can bring the charges to the target shape and area and therefore form the desired deformation. As commonly accepted, the electrode materials should have some properties: (1) They have the ability to maintain conductivity during large strains; (2) their stiffness can be ignorable, comparing with that of DEM; (3) they have the ability to maintain good stability [49]; (4) they are preferably to be patternable for conducting flexible electrode designs [50]. For applications on cells and tissues, as reported by Samuel Rosset et al. [51], manufacturability, miniaturization, impact on DEA performance, and the compatibility with low-voltage operation need to be taken into consideration. In this section, some widely used electrode materials are introduced.

#### Carbon-Based Electrodes

Because of the low stiffness and ability to maintain conductive at large strain [50–52], carbon-based electrodes are the most popular electrode materials for DEAs; typically, they can be divided into three main categories: carbon powder, carbon grease, and conductive rubber.

**Carbon Powder Electrodes** The main outstanding merit of powder-based electrodes is their less contributory to the stiffness of the DEM. Applying the loose carbon powders directly on the membrane became the solid choice in the early stage. However, the disadvantages of carbon powder are obvious: It is difficult to maintain conductivity at large strain [53, 54] and lifetime is also limited because of the detaching of conductive particles from electrodes [51].

**Conductive Rubber Electrodes** Similar but not identical to carbon grease, conductive rubbers are produced through directly mixing conductive particles with silicone. As a result, the ablation or migration of the conductive particles can be avoided, and the lifetime of the electrodes can be extended. However, the impact on the stiffness of the DEM is not negligible [51].

**Fig. 1:**
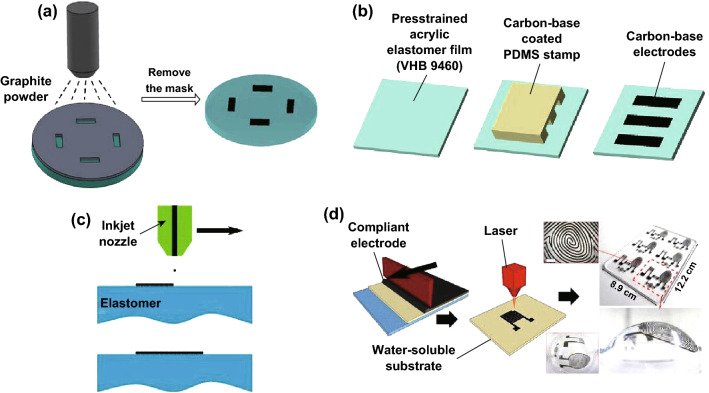
The techniques for creating carbon-based compliant electrodes. **a** Shadow masking: using a shadow mask to selectively spray the carbon material on target area and then removing the mask to get the final electrodes. **b** Stamping process: using patterned elastomeric stamp to pick up the carbon material and stamping it on the DEM. Redrawn from Ref. [56] with permission. **c** Printing: the carbon-based materials can be made into conductive ink; then using printing technology to pattern electrodes. Adapted from Ref. [51] with permission. **d** Laser ablation: the thick PDMS–carbon composite layers can be patterned by laser ablation and bonded to PDMS membrane by oxygen plasma activation. Adapted from Ref. [58] with permission

With less requirements on precision, thickness homogeneity, and shape of the electrodes, they can be easily painted on the DEM. Nevertheless, for cellular research, the DEAs usually command accurate electrode pattern. Here, we introduce several techniques to precisely fabricate the carbon electrodes on the DEM as shown in Fig. 1.

Clearly, a mask covered on the surface of the membrane can be helpful to paint the carbon material into desired shape. Pelrine et al. [54] have presented their work for fabricating loose carbon grease and powder-based electrodes. To improve the uniformity, Schlaak et al. [55] proposed the use of spray coating as presented in Fig. 1a, which is an efficient manufacture method and can be used for commercial applications. Similarly, the electrodes can be stamped on the DEM [56]. In such case, fabricate a soft stamp into desired pattern by replication on an etched silicon negative master as shown in Fig. 1b. Besides, printing techniques can also be used to pattern electrodes as presented in Fig. 1c since the carbon-based materials can be made into conductive ink [51]. As reported by Krebs, printing methods have been utilized to manufacture flexible devices [57]. More advanced, high-resolution and robust compliant electrodes for silicon-based soft actuators and sensors are completed through laser ablation technology [58] (Fig. 1d).

#### Metallic Electrodes

**Fig. 2:**
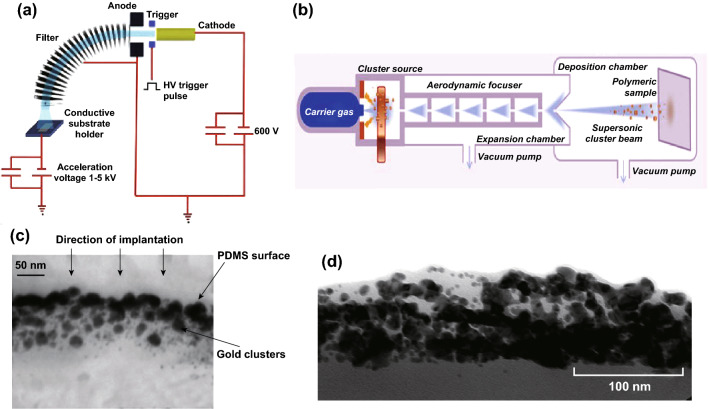
**a** The schematic picture of FCVA. Adapted from Ref. [59] with permission. A high-voltage (600 V) impulsion initiates the main arc from the cathode, the filter helps to trap the macroparticles and the negatively substrate holder accelerates the positive ions through the plasma sheath. **b** The schematic of SCB progress. Adapted from Ref. [63] with permission. The Au NPs nanoparticles generated from the cluster source is accelerated by a carrier gas in a supersonic expansion and then focused by aerodynamic lens. The cluster beam can be injected into the deposition chamber and impacted on the surface of a thermo-retractable polystyrene (PS) sheet. **c** The product of FCVA. Adapted from Ref. [47] with permission. **d** TEM image of the product of SCB. Adapted form Ref. [63] with permission

Due to the low resistance, metallic electrodes are also alternative for the field of DEAs. However, the high Young’s modulus and relative small strain make it difficult to directly pattern them. To overcome this drawback, some fabrication methods have been proposed, such as photolithography, depositing [51], and implantation. Among these methods, DEAs with implanted metallic electrodes present better performance. Here, we introduce two methods to implant metallic electrodes: filtered cathodic vacuum arc implantation (FCVA) and supersonic cluster beam (SCB) implantation. The principle of FCVA can be depicted simply [59–62]: The plasma generated from the source consists of metal ions, electrons, and the undesirable macroparticles, and the magnetic filter is used to remove the macroparticles from the plasma; nanometer-sized clusters can be generated through this technology (Fig. 2a). Similarly, the progress of SCB is shown in Fig. 2b: Vaporize the metal firstly and then use a pulse of inert gas to quench the plasma to form the neutrally charged clusters. Then, the clusters can be injected into the deposition chamber and form metal/PDMS nanocomposite layer. Figure 2c, d shows the nanostructure of the metallic electrodes that are produced via FCVA and SCB, respectively.

#### Transparent Electrodes

Basically, the conductive materials which meet the requirements of DEA electrodes application are non-transparent. However, some works proposed several transparent electrodes that can be potentially used in optical applications. As reported, Hu et al. [64] have studied transparent and conductive nanotube thin films on electrical and optical properties. Kovacs et al. [65] found that loose carbon black with extremely thin thickness can be partly transparent when applied on adhesive acrylic membrane. Implanted palladium and gold electrodes have also been reported to be possibly present transparency with a ratio of 35 and 70%, and the value is dependent on the metal and the implanted dose [62]. Besides, ionic hydrogel is a novel, ordinarily, and transparent material for electrodes of DEAs [66, 67]. Combining ionic hydrogel with 3D printing, people successfully fabricated electric-driven soft actuators that can achieve a maximum vertical displacement of 9.78 ± 2.52 mm at 5.44 kV [68].

As mentioned above, DEAs for cellular research need to be optically transparent. Therefore, the development of transparent electrodes can accelerate the applications of DEAs of cellular use; before that, much work is still demanded to develop such novel electrodes materials and techniques.

## Actuation Mechanism of DEAs

**Fig. 3:**
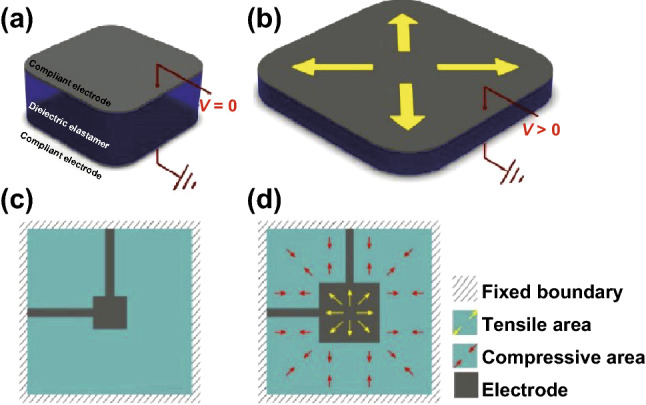
The working principle of dielectric elastomer actuators (DEAs) and the classical DEAs for cellular mechanical stimulus. Adapted from Ref. [71] with permission. **a** The device consists of a soft dielectric elastomer sandwiched between compliant electrodes on both top and bottom sides; the actuator keeps static without voltage applied, and **b** the device is motivated when the voltage is applied. Similarly, for cellular mechanical loading, the DEA **c** keeps the original state without HV applied and **d** deforms underactuated and then generates two regions as tensile strain area and compressive area

In general, DEA is a device that converts electrical energy into mechanical deformation [28]. The basic actuation mechanism is presented in Fig. 3, a dielectric elastomer film is sandwiched by the patterned electrodes on both top and bottom sides, and high-voltage (HV)-induced compression along thick direction causes in-plane expansion. This physical response of dielectric elastomers can be linked with their Maxwell stress effect [49]. Since the electrode patterns can be various, the specific strain characterization of DEAs can be different in the concrete cases. Typically, the strain along the thickness direction *S*_M_ [69] and the effective electromechanical pressure *P* [70] of the elastomer membrane can be used to describe the strain level.

1 $$S_{\text{M}} = - s\varepsilon_{0} \varepsilon_{r} E^{2} /2$$

2 $$P = \varepsilon_{0} \varepsilon_{r} E^{2}$$

where $\varepsilon_{0}$ is the permittivity of vacuum, $\varepsilon_{r}$ is the dielectric constant of the elastomer, *E* is the electric field applied, and *s* is the elastic compliance.

Generally, for the DEA-based devices, it is noteworthy that the pre-stretch of DEM is another indispensable design parameter that can affect the performance. Obviously, pre-stretching can prevent the buckling of the dielectric elastomer when electrically activated. Secondly, pre-stretching the elastomer contributes to improve the performance of DEAs since it can increase electromechanical instability (EMI) of the membrane [28, 72–75]. Additionally, equiaxial and uniaxial pre-stretch can help to generate equiaxial and uniaxial strain, respectively. Therefore, it is common to change the amplitude and ratio of pre-stretch to generate desired behaviors of DEAs.

Figure 3c, d shows the classical configuration of DEAs for cell mechanical stimulus [71]. Usually, the rigid frame is necessary to hold pre-stretch of the DEM. Once the voltage is applied, the area with electrodes is expanded, while the remaining area is compressed. As a result, the expanded area is defined as active and can be used for stretching the cells and the compressed area is called passive and can be adopted to compress the cells.

## Evaluation of DEAs

It is important to understand cellular environment when studying cells’ responses to the mechanical loadings, so precisely characterization of DEA devices is indispensable, that is, we need to gain the strain distribution of our target area in the DEA. Currently, finite element modeling (FEM) and image processing (machine vision and digital image correlation) techniques are widely used to calculate the strain distribution.

FEM is a non-contact way commonly used in many fields to calculate stress and strain distribution of a mechanical/structural system [76]. To complete the FEM, some basic parameters of the materials are required such as Poisson’s ratio and elastic modulus. Constrain the model with certain boundary conditions and divide it through multiple grids, calculate the strain in every single grid, and finally generate the whole strain distribution. For example, Akbari et al. [47] optimize the geometric configuration of the actuator by using a simplified FEM.

**Fig. 4:**
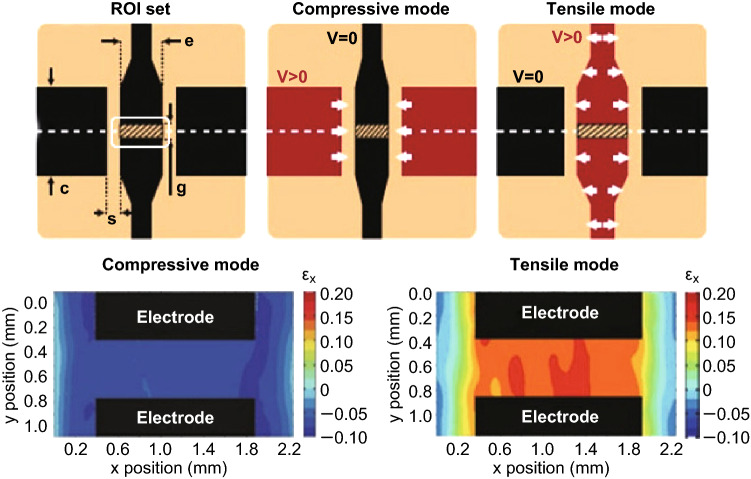
Example of characterizing DEA system via DIC. The white rectangle is region of interest (ROI) set. Strain distribution of both compressive and tensile mode can be obtained through DIC. Adapted from Ref. [78] with permission

Image processing technology can be helpful as well and seems more popular among the people who study cells via DEAs. Similar to FEM, digital image correlation (DIC) is another non-contact method to obtain the strain distribution. Set the region of interest (ROI) on the original image; the algorithm conducts correlation calculation in the image after deformation to find the most relevant points/pixel with the original one, and then, displacement and strain can be calculated. Recently, Blaber et al. [77] published their open-source 2D digital image correlation software. With this software, the strain distribution of the actuator can be successfully measured by processing the pictures in actuated and rest state. For instance, as shown in Fig. 4, Poulin et al. [78] obtained the strain distribution at the ROI from both compressive and tensile modes through DIC. Analogously, it is easy to complete the characterization when the shapes of electrodes changed [45].

Besides the strain distribution, the basic deformation–voltage responses (average strain) can be obtained through image processing as well. As reported by Rosset et al., they measured the strain of actuator using machine vision via a LabVIEW image processing to track the four corners of the electrodes [79]. After calibration, the coordinates of these four corners that are under both actuated and non-actuated states are recorded, so that the curve of voltage induced strain can be plotted.

## DEA-Based Devices for Cellular Research

According to the different cell amounts that the DEA-based bioreactors may apply in, they can be divided into two categories: one for single cell and the other for a small population of cells or in other word tissue engineering.

### DEA-Based Bio-Stretcher/Reactor for Single Cell

**Fig. 5:**
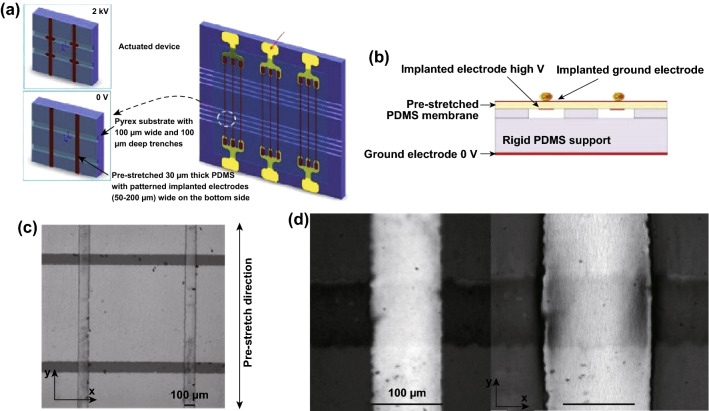
The schematic of DEA designed for single-cell mechanical stretching. **a**, **b** The micro-actuator arrays are designed for single cell; each intersection of vertical lines and horizontal lines forms an individual actuator, where cells can be stretched. Adapted from Ref. [47] with permission; **c** top view of a small part (four micro-actuators contained) obtained by microscope; the membrane is pre-stretched along y-axis. The bright lines aligned vertically are the ion-implanted electrodes on the top side of PDMS membrane, while the dark lines aligned horizontally are the bottom electrodes. Adapted from Ref. [46] with permission. **d** Micrograph of one actuator. Adapted from Ref. [84] with permission

For single-cell mechanical stimulation, technologies like microfluidics [80], atomic force microscope [81], microelectromechanical systems [82], and optical tweezers [83] have been widely used. The DEA-based devices for single-cell mechanical loading rely on the principle that cells may deform with the stretchable substrate where they already adhere. Now, such devices are at the stage of conceptual design. Figure 5 shows the schematic of DEA designed for stimulating single cell. Implanted gold ion electrodes are patterned on both sides of PDMS film [47]. Continuous electrodes are implanted on the top side of membrane, while the bottom side has narrow implanted electrodes. Red lines are ion-implanted electrodes on the bottom of a PDMS membrane and horizontal lines are trenches with square cross section. Mechanical stretch happens in the intersection regions of the top and bottom electrodes. Such design forms numerous units, and Fig. 5c actually shows four units for four cells to be stretched [46]. When the DEA is actuated by a voltage of 3.8 kV, the membrane expands by 56% along the x-axis (Fig. 5d).

### DEA-Based Bioreactor for Small Population of Cells

**Fig. 6:**
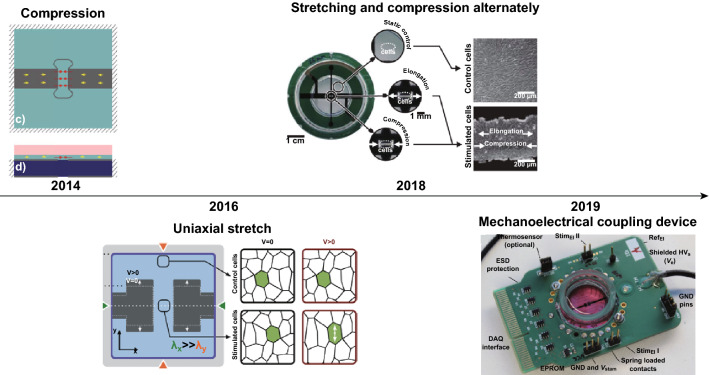
The DEA devices for cellular stimulations. Uniaxial compression was proposed by Alexandre et al. in 2014, which harnesses stress in passive region of the DEA and stimulates the cells on top of the region. Adapted from Ref. [71] with permission. In 2016, they fabricated the DEA for uniaxial stretching of cells, and LECs were used to test the device. Adapted from Ref. [44] with permission. Poulin et al. proposed the active cell culture substrate which can produce complex strain patterns with extremely high-strain rates in 2018. Adapted from Ref. [78] with permission. In 2019, more advanced DEA-based bioreactor providing mechanoelectrical coupling stimulus is proposed. Adapted from Ref. [85] with permission

For a small group of living cells, it is more flexible to generate tensile or compression strains through DEAs, and the corresponding research is shown in Fig. 6. In 2014, Alexandre et al. [71] reported that the stress in passive region of DEAs can be utilized to compress the cells. Since then, in 2016, they developed the DEA-based cell stretcher to stimulate lymphatic endothelial cells (LECs) and demonstrated that DEAs can be interfaced with living cells and used to supply mechanical loading [44]. After that, an innovative muscle-like bioreactor (mimicking the small intestinal) for the investigation of physiological phenomena was described by Cei et al. [70]. The bioreactor can maintain its performance even if incubated with Caco-2 cells for 21 days until the differentiation of cells can be observed. In 2018, the actuator that can generate alternately tensile and compression strains was proposed by Poulin et al. [78]. Afterward, as the newest work, for the purpose of investigating drastic cases like rapid stretch effect on cardiac tissue, Imboden et al. [85] showed their high-speed mechano-active multielectrode actuator, which can provide stimulus of mechanoelectrical coupling mechanism.

Such a progress actually indicates the diversified future of the DEA-based bioreactor. DEAs can be applicable for different purposes and further fabricated into the bioreactor with a specific function.

### DEAs Designed for the Measurement of Traction Force of Cells

**Fig. 7:**
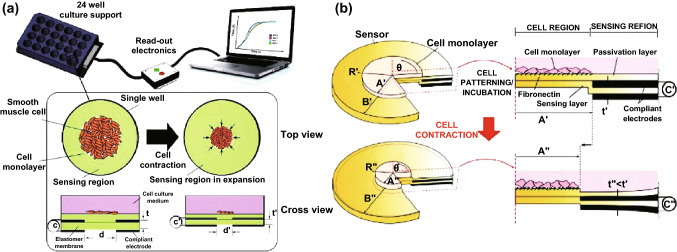
**a** Schematic representation of the measure system proposed by Araromi et al. in 2015: The 24-well cell culture supports are equipped with integrated strain and force measurement sensors that are based on DEA. Besides, the electrical read-out allows for high-throughput parallel measurement in real time. Adapted from Ref. [91] with permission. **b** Optimization of the DEA-based sensors. Parameters such as *R*, *θ*, *A*, and *B* are used to model the system. Adapted from Ref. [92] with permission

Monitoring the biological indicators of cells, e.g., metabolic analysis, biomarker detection, cell force, and strain, is very necessary for investigating the cells’ behaviors and understanding some diseases. The metabolic analysis and biomarker detection mainly rely on mass spectrometry and electrochemistry ways [86–88], while force sensing technologies predominantly rely on optical methods [89], which hinders scaling up of devices for the purpose of parallelized and real-time measurements. Except for the function of passing mechanical loading to the living cells, as reported, the DEAs can also be the potential sensors to measure traction force of cells. For example, in 2017, Rosset et al. [90] used the DEAs to achieve subcellular resolution measurement of cell traction forces. A DEA-based sensor system for measuring the contraction force of smooth muscle cells was also reported [91], and the principle is shown in Fig. 7a: The system contains three main parts, including the DEA-based cell culture support (24-well unit is designed for high-throughput parallel measurement), the read-out electronics and the computer to show the measured data. Actually, every culture well works independently to sense the expansion which is caused by cellular contraction and the changes in the device capacitance which is caused by the contraction. Cell contraction causes changes in the geometry, i.e., diameter of the cell region and thickness of the DEM from *d* to *d*′ and *t* to *t*′, respectively. These changes consequently cause changes in the device capacitance that can be measured.

In 2016, the same group proposed further optimization of thin-film sensors for contractility measurement of muscle cells [92]. In this work, they proposed their modeling to predict sensor behavior as shown in Fig. 7b. They describe the system based on cylindrical coordinates which use *R* and *θ* to model the in-plane information and *Z* to present the thickness information. The final capacitance after cell contraction can be expressed as Eq. 3:

3 $$C^{{\prime \prime }} = \varepsilon_{0} \varepsilon_{r} \int_{A}^{B} {\frac{{2\pi R{\text{d}}R}}{{t\lambda_{Z}^{2} (R)}}}$$

where *R* is the radial position of an arbitrary point in the sensing region before pre-stretch and cell contraction, *t* is the initial sensing layer thickness before pre-stretch, and $\varepsilon_{0}$ and $\varepsilon_{r}$ are the permittivity of free space and the relative permittivity of the sensing layer, respectively. *A* is the radius of cell region before pre-stretch, *B* is the radius of the DEM before pre-stretch, and *λ*_*Z*_ is the stretch in the thickness direction (assuming material incompressibility).

## Comparison Between Usual Commercial Bioreactors and the DEA-Based Ones

**Fig. 8:**
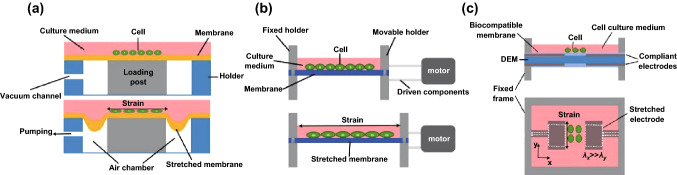
Schematic of typical pneumatic, motor-driven, and the DEA-based device. **a** Side view of classic pneumatic cell stretching devices. An air chamber is formed by combining the membrane, holder, and loading post. Pumping air from the chamber can generate pressure difference that leads to absorption of membrane and stretch the cells. **b** Side view of motor-driven stretching devices. The membrane with seeded cells is matched with two holders: One is movable, while the other is fixed. The movable holder connects with motor through driven components (gears, track, etc.). Once control signal input to the motor, generated motion drags the holder, causing stretching strain as consequence. **c** Side and top views of DEA-based cell stretcher. The DEA is sandwiched by biocompatible membrane and then fixed by rigid frame

In this part, we provide comparison between the widely used commercial devices and the DEA-based ones. The traditional mechanical systems can be typically divided into two categories, the motor driven (STB series from STREX) and the pneumatic (FX series from Flexcell). Generally, almost all of the mechanical methods to stimulate cells in vitro rely on a membrane (usually PDMS or silicone that are biocompatible) to deliver the stimulus. For the pneumatic devices, the membrane is placed on the holder, a loading post is filled to form the air chamber, and a channel is made to allow the operation of pumping (Fig. 8a). Once we pump air out from the chamber, the atmospheric pressure will squeeze the membrane into the chamber and consequently create a tensile strain. In contrast, motor-based system is more direct. The membrane (or chamber) is installed on two holders. One is fixed, while the other is movable and is connected with the motor through driven components. When activate signal is applied, the motor can generate corresponding rotation; the driven components then translate the rotation to traction of the movable holder for stretching the membrane (Fig. 8b). For the DEA-based bio-stretcher, uniaxial pre-stretch of DEM usually is set to produce the strain along one desired direction. Biocompatible membrane in which cell is cultured firstly is placed on the DEA after electrodes patterning, and then rigid frames are used to fix the membranes. The compliant electrodes will expand along the reverse direction of pre-stretch; once HV is applied, then cells can be stretched (Fig. 8c). Usually, the volume of the pneumatic and motor-driven devices is higher than that of the DEAs, because of the extra elements such as air chambers and movable holders. Besides, the pneumatic and motor-driven methods require the pump and motor to activate the membrane; in other words, these are indirectly control system, facing problems that strain can be limited since the original activated signal which need to be converted may beyond the performance of the pump or motor. In contrast, DEA-based devices are motivated directly by the electric signal, which is another unique advantage.

**Fig. 9:**
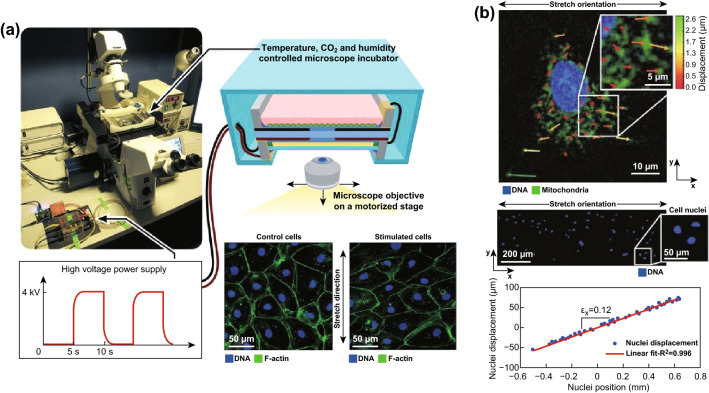
Real-time monitor system through DEA devices. **a** Schematic of the whole system with DEA-based cellular stretcher and inverted microscope. The DEA is motivated and controlled by the high-voltage source, a mini incubator is used to provide the standard environment for living cells (lymphatic endothelial cells (LECs) used), and the microscope is used to monitor the cells. Adapted from Ref. [44] with permission. **b** Imaging of lung cells A549 during stretching: The DNA and mitochondria are stained as blue and green, respectively. Top picture shows displacement track of the intracellular content of A549 at ×40 magnification; the arrows with different colors and lengths refer to different degrees of displacements. Bottom picture shows the nucleus track of A549, *ε*_x_ = 0.12 along the stretch orientation measured through this method, and a line is found to fit the nuclei displacement perfectly. Adapted from Ref. [78] with permission

When it comes to specific using, several parameters may be important to assess the devices, including available frequency and strain. Here, we obtain the information of several well-known commercial motor-driven and pneumatic systems by searching their manual on the Web site [93, 94] and list them in Table 1. The performance of the pneumatic highly depends on the membrane and the vacuum pump, while for motor-driven devices, the key element is the motor itself. As mentioned above, DEAs are directly activated via electric signal without the progress of conversion from control signal to motor driver or vacuum pump that can restrain the performance, i.e., the faster response time and higher frequency may be obtained. Thus, DEA-based devices can theoretically provide more various loadings than the other two ways. In addition, due to the compact volume and highly flexible design of DEAs, real-time monitoring of cells via microscope is feasible and relatively simple. For instance, Poulin et al. [44] presented the DEA-based real-time monitor cellular stimulating system (Fig. 9a). The cell-seeded DEA device is placed in a simplified transparent incubator over the microscope objective, and the microscope is programmed to periodically capture the cell picture. In addition, for clear observation of cellular internal elements like nucleus under mechanical stimulus, staining is also compatible in such system. They stained the human lung carcinoma cells A549 and recorded the dynamic position of cellular DNA and mitochondria during uniaxial stretch. The nuclei displacements they measured show a linear relation with the initial nuclei positions, which can be the evidence that DEA-induced strain is transferred to the cells [78].

**Table 1:** Performance parameters of different technologies and models

Technology	Model	Strain	Frequency	Signal wave	Microscope used
Motor-driven [93]	STB-1400-04	Uniaxial stretch 2, 4, 5, 8, 10, 12, 15, 20%	1/60, 1/30, 1/10, 1/6, 1/3, 1/2, 1 Hz	64 patterns	–
	STB-1400-10	Uniaxial stretch 2, 4, 5, 8, 10, 12, 15, 20%	1/60, 1/30, 1/10, 1/6, 1/3, 1/2, 1 Hz	64 patterns	–
	STB-150	Uniaxial stretch 2, 4, 6, 8, 10, 12, 15, 20%	1/60, 1/10, 1/3, 1 Hz	64 patterns	Nikon and Olympus
	STB-150w	Uniaxial stretch (2 switchable modes)	1/6, 1/3, 1/2, 1 Hz	64 patterns	Nikon and Olympus
	STB-190-XY	Biaxial stretch and compression, ~ 30%	0.05, 0.2, 0.5 Hz	64 patterns	Nikon and Olympus
Pneumatic [94]	FX-5000	Stretch, ~ 30%	0.01–5 Hz	Sinusoidal, etc.; custom definable	Upright microscope
	FX-6000	Compression	0.01–5 Hz	Sinusoidal, etc.; custom definable	Upright microscope
DEA-based [78, 85]		Uniaxial stretch ~ 38% compression ~ 12%	> 10 Hz, customer definable	Customer definable	Inverted microscope

STB, the motor-driven device provided by STREX; FX, the pneumatic device provided by Flexcell

In a word, DEAs can be a greater available carrier choice for cellular mechanical loading research. Therefore, we give a brief outlook of DEAs’ applications (next section) and hope to enlighten the combination of mechanobiology and DEAs.

## Outlook of DEAs in Cellular Mechanical Loading Research

A phenomenon of cellular and tissue behavior adjustment is strongly associated with the changes in extracellular matrix (ECM) and protein expression, which can be determined by the loading and cell type. For in vitro cellular stimulus, we can deform the cell membrane by stretching the cell adhesion substrate [95, 96]. Over the past years, people have conducted interesting researches on the load-sensitive cells, trying to discover the relations between cellular responses and different loading conditions. Although most of the published results were completed by the pneumatic and motor-driven devices, comparison suggests that DEA-based devices share the properties as well or even better, so the following applications can theoretically be the references for development of DEA-based bioreactors.

Among the various cellular responses, reorientation of the cells is intuitively visible, and the relevant research has been carried out for a long time. In 1986, Dartsh et al. [97, 98] presented their work to cyclic stretch smooth muscle cells, and the result shows uniform reorientation of the uniaxial stretch cells compared to the control group. Then, experimental results suggest that cells form weak adhesions on the soft substrates [99], which allow the reorientation under in vitro mechanical loading. Based on these results, people tempted to model this phenomenon. They proposed that mechanical sensor system of cells is the reason why cells response to the loadings; they mainly focus on the state dynamics of cells during reorientation such as focal adhesions (FAs), stress fibers (SFs), and actin cytoskeleton. Some representative theories were raised. For example, a widespread theory once proposed that cells realign along the zero (or minimal) strain direction to maintain original undisturbed state [100], and the theory can be expressed as Eq. 4:

4 $$\theta = \arctan \left( {\sqrt { - \frac{{\varepsilon_{{\text{xx}}} }}{{\varepsilon_{{\text{yy}}} }}} } \right)$$

where $\varepsilon_{{\text{xx}}}$ and $\varepsilon_{{\text{yy}}}$ refer to the strain along x and y directions, respectively. However, Livne et al. [8] found deviation between the experimental cellular reorientation and theoretical predictions above and proposed the new theory that regards the reorientation as result of dissipative process to relax the passively stored elastic energy, and can be predicted as Eqs. 5 and 6:

5 $$\theta = \arccos \left( {\sqrt {b + \frac{1 - 2b}{r + 1}} } \right) = \arctan \left( {\sqrt {\frac{r + b \cdot (1 - r)}{1 - b \cdot (1 - r)}} } \right)$$

6 $$r = - \varepsilon_{{\text{yy}}} /\varepsilon_{{\text{xx}}}$$

**Fig. 10:**
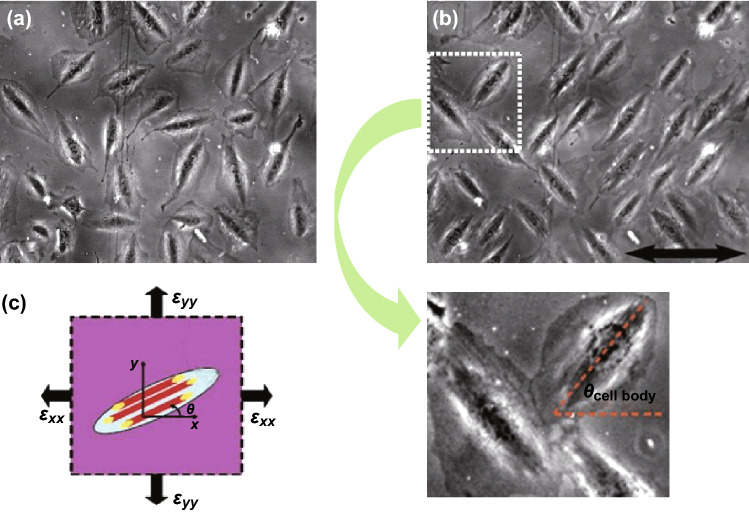
Reorientation of the cells. **a** Cells (REF-52 fibroblast) with random orientations before stretching. **b** Reorientation after stretching. **c** The description of strain reference on a polarized cell. Adapted from Ref. [8] with permission

This function is valid for *r* ∈ [1 − 1/*b*, 1 + 1/(*b −* 1)], where *b* is a dimensionless parameter which is related to cellular Young’s moduli along the polarized reference system (Fig. 10). For verification, they tracked cells under various original angles and stretch parameters and obtain well fit between the experimental results and the predicts. More recently, Chagnon-Lessard et al. [101] demonstrated that strain gradients guide the orientation as well. The mechanism was described as gradient avoidance response, and the statistic results show great similarity of cellular arrangement between high-strain region and the low-strain but high-gradient area.

**Fig. 11:**
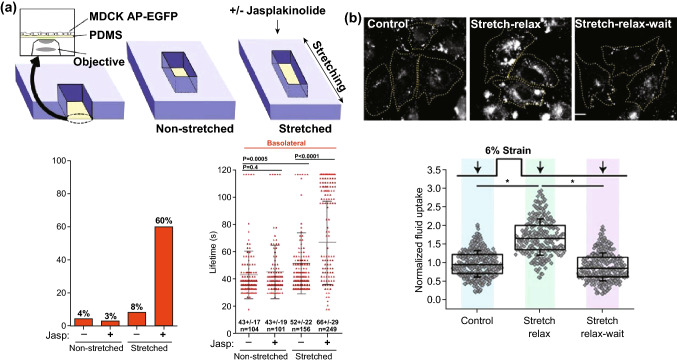
Effect of membrane tension differences on cellular endocytosis. **a** Jasplakinolide treatment increases pit lifetime and percentage of arrested pits of stretched cells, adapted from Ref. [104] with permission. **b** Stretching regulates F-Dex uptake, adapted from Ref. [105] with permission

Besides, some basic functions of cells, such as endocytosis and exocytosis, are associated with the membrane tension. In fact, the decrease in tension improves endocytosis, while an increase in tension brings suppression [102, 103], and works have been conducted to research such endocytosis modulating through mechanical stimulus. For example, Boulant et al. [104] used a mechanical stretching device (motor-driven) to stretch the cell substrate to verify the adjuvant effect of actin- on clathrin-mediated endocytosis. They found that jasplakinolide treatment of the stretched cells causes a dramatic increase in pit lifetimes and percentage of arrested pits under 25% stretching, which shows that the clathrin-mediated endocytosis must be assisted by actin in order to complete the vesicle dissociation process under high membrane tension. Thottacherry et al. [105] applied 6% stretch strain to the Chinese hamster ovary (CHO) cell and observed a remarkable reduction in fluorescent dextran (F-Dex) endocytosis compared to the static. However, F-Dex uptake increases significantly at the movement of stretch relax (Fig. 11b); they thus proposed the CLIC/GEEC(CG), a dynamin-independent pathway that can react to the change in membrane tension and regulate F-Dex uptake.

For broader fields to discuss, tumor research and rehabilitation engineering may be appropriate [106–108]. Uncontrolled proliferation of tumor causes forces interaction to the ECM and tissues nearby, which can be usually classified into shear, compress, and stretching stresses [106]. Hofmann et al. [109] found that mechanical stretching increases the proliferation of cancer cells, while Helmlinger et al. [110] found that compressive stress inhibits the growth of tumor spheroids. Besides, cells and tissues in human’s joint undergo complex forces during our daily life, which means that rehabilitation research from athletic injury can be inspired from mechanical stimulus as well. As reported, the state of tendon and meniscus can be associated with mechanical loadings. 10%-strain dynamic compression promotes anabolic of meniscus, while strain at 20% regulates the state into catabolism [111], and the responses can be frequency and time dependent. More interestingly, cyclic tension strains show inhibition of inflammatory of meniscal cells [107]. In addition, experimental results demonstrated that tendons also respond to mechanical loads, appropriate loads enhance tendons, while chronic mechanical loading may accelerate tendinopathy [112, 113].

In this section, we simply introduce several potential applications of DEA-based devices for mechanobiology research, including cellular reorientation, endocytosis, etc. Actually, much more concrete scenarios of this field are still waiting to be explored. Similar to the motor-driven devices, DEAs are at the junction of medical (biology) and engineering science; they can be designed into various mechanical loading bioreactors while maintaining cell and tissue affinity. As a promising tool, we hope that DEAs can contribute to the development of mechanobiology.

## Conclusions

Exploring the response of cells is an exciting, evolving but challenging task which is significant for biomedicine engineering. Directly, many illnesses can be linked with the disordered cells and tissues functions, which means that it makes sense to research cellular responses under various mechanical loadings for better understanding of some diseases or even cancers. Furthermore, if we can regulate cells and tissues into the proper states or functions through mechanical stimulus, some effective and promising treatments can be developed.

In this work, we firstly provide simple introduce of DEAs, including components, actuation principle, evaluation methods, and several applications on cellular mechanical loading. Then, we compare the DEA-based bioreactors with current widely used custom-built bioreactors, showing their connections and differences, and some prominent properties of DEAs stand out. At last, we give short outlook of DEA technology in the future mechanobiology research.

In a word, although much corresponding examples are still lack, employing DEAs as the bioreactors and biosensors for cellular applications is actually opening the door of cellular mechanobiology through a novel method. As the new generation of actuators, DEAs bring some irreplaceable advantages compared to traditionally used peers like the motor-driven and pneumatic: They have simpler structure, faster response, and higher controllability. In addition, DEAs are more flexible to design and can be easily catered the request of biocompatible and combine with microscope to form an experimental system. Among these advantages, the property of rapid response makes the DEA-based devices potential to simulate some extreme conditions, such as sudden cardiac death, which is absolutely difficult to realize by some other bioreactors. Furthermore, continuous advances in material science and microfabrication technology make it feasible and promising to study cellular response of mechanical stimulus through DEA devices since they can be manufactured into micro–nanoscale, and then design into high-throughput devices that are meaningful for cellular research. What is more, because the using of powerful algorithm and image processing tools, this field can be multidisciplinary and a hot issue in the future, which means low threshold for the people to conduct this study.

Nevertheless, some challenges still remain elusive. Firstly, the drive voltage for DEAs is usually too high (several thousand volts), which makes this technique risky and limit their broad applications. Therefore, much works are still need to cut down the required voltage or electric field. As reported, for example, Shea’s group have tried to reduce the voltage by decreasing the thickness of DEM [114]. Secondly, optimization of DEAs’ basic performance can be crucial, including larger strain, higher energy density, longer lifetime (cycles that can be tolerated, longer shelf time), and better stability, and all of these are important to determine DEAs’ further applications in both cell and tissues’ mechanobiology and other possible fields.
